# Evaluating the Impact of Air Quality on Pediatric Asthma-Related Emergency Room Visits in the Eastern Province of Saudi Arabia

**DOI:** 10.3390/jcm14134659

**Published:** 2025-07-01

**Authors:** Abdullah A. Yousef, Reem Fahad AlShammari, Sarah AlBugami, Bushra Essa AlAbbas, Fedaa Abdulkareem AlMossally

**Affiliations:** 1 Department of Pediatrics, College of Medicine, King Fahd Hospital of the University, Imam Abdulrahman Bin Faisal University, Dammam 31441, Saudi Arabia; aaayousef@iau.edu.sa; 2 College of Medicine, King Fahd Hospital of the University, Imam Abdulrahman Bin Faisal University, Dammam 31441, Saudi Arabia; alabbasbushra@gmail.com (B.E.A.); ffeeddaa6@gmail.com (F.A.A.); 3 Department of Physics—GIS Program, College of Science, University of Jeddah, Jeddah 23890, Saudi Arabia; saalbugami@uj.edu.sa

**Keywords:** pediatric, asthma, air pollution, emergency department visit, air quality, environmental triggers

## Abstract

**Background/Objectives**: Pediatric asthma is a leading cause of emergency department visits, and air pollution is a known primary environmental trigger. Although worldwide air pollutants have been associated with asthma exacerbations, limited data have been reported in the Eastern Province of Saudi Arabia. This study aimed to investigate the relationship between air pollution and pediatric asthma admissions among children aged 2 to 14 years old at King Fahd Hospital of the University Hospital (KFHU). **Methods**: This is a retrospective cohort study, over 366 days, including 1750 pediatric asthma-related ER visits and daily concentrations of air pollutants (PM_2.5_, PM_10_, NO_2_, SO_2_, CO, and O_3_) and meteorological factors (temperature and humidity). Various statistical models, such as Poisson regression and ARIMA, were applied to determine the association between pollutants levels and hospital ER visits. The data were visit-based in nature, and it was not possible to follow up with repeat visits or for admission status for individual patients. **Results**: Elevated levels of PM_2.5_, NO_2_, and CO were significantly associated with more pediatric asthma ER visits, mainly on the same day and with short lags. PM_2.5_ displayed the strongest association, consistent with its deeper pulmonary penetration and greater toxicity. Also, PM_10_ levels were inversely associated with ER visits, possibly due to particle size and deposition location differences. Significantly correlated with increased ER visits are lower ambient temperature and higher humidity. **Conclusions**: This study offers strong evidence on the relationship between air pollution and pediatric asthma events, in turn highlighting the vital importance of air quality regulation, public health policies, and clinical vigilance for environmental exposures.

## 1. Introduction

Asthma is a common chronic respiratory disease characterized by bronchial hyperresponsiveness and airway inflammation that leads to symptoms of wheezing, shortness of breath, and coughing. It is among the most common chronic conditions in the world, and it is particularly common in children. According to the Centers for Disease Control and Prevention (CDC), approximately 1 in 13 people in the United States have asthma [1]. Although it can occur at any age, it usually presents in childhood and is a common reason for emergency department visits.

By 2025, asthma will rank among the most prevalent chronic non-communicable diseases, affecting over 260 million people and contributing to over 450,000 annual deaths globally [2]. The prevalence of physician-diagnosed asthma in children in Saudi Arabia has been reported to vary between 4% and 33.7%, with the highest rates observed in Al-Hofuf and Najran [3]. The prevalence and severity of asthma have been increasing due to industrialization, urbanization, and environmental changes. Climate change, high levels of greenhouse gas emissions, and fine particulate air pollution have been identified as contributing to the exacerbation of respiratory diseases, in particular, asthma [4].

Fine particulate matter (PM_2.5_), nitrogen dioxide (NO_2_), sulfur dioxide (SO_2_), and ozone have been associated with enhanced airway inflammation, increased sensitivity of the airways, and more severe attacks of asthma. Long-term exposure to these pollutants has been linked to a decline in lung function and increased risk of emergency room visits [5]. Moreover, air pollution can also lead to oxidative stress and immune dysfunction, resulting in further worsening in respiratory symptoms. Children are even more susceptible because they have developing lungs, along with a relatively high ventilation rate and absorbing capacity, thus making them susceptible to inhaled toxins [6].

Several studies, including those of exposure to Asian dust, have reported strong associations between air pollution and deterioration of asthma symptoms in non-asthmatics [6,7]. These particles may act as acute triggers for asthma attacks, especially in children living in urban or industrial areas [8]. Despite the global focus on the respiratory effects of poor air quality, there remains a substantial knowledge gap in the Middle Eastern region, particularly in Saudi Arabia, where few studies have specifically studied the relationship between air pollution and pediatric asthma-related emergency visits.

The objective of this study is to assess the association between short-term exposure to air pollution particles, including particulate matter originating from recurrent dust storms in Saudi Arabia and the rate of emergency department (ED) visits of children aged 2 to 14 years diagnosed with asthma in the Eastern Province, Saudi Arabia. This study aims to establish evidence by combining data on air quality with hospital-visit data, which may support public health strategies, and clinical measures to reduce the burden of asthma exacerbations among this high-risk population.

## 2. Materials and Methods

### 2.1. Study Design and Setting

This is a retrospective cohort that was performed at King Fahad Hospital of the University (KFHU), a tertiary-care facility in Al-Khobar, Eastern Province, Saudi Arabia. Our aim was to assess the relationship of air pollution levels and pediatric asthma-related emergency room (ER) visits in a 1-year study (from July 2023 to July 2024). Ethical approval was obtained from the Imam Abdulrahman bin Faisal University Institutional Review Board (IRB; Approval Number: IRB-2024-01-603), granted on 3 September 2024.

It should be noted that the data were obtained on the visit level, and individual identifications of patients were not available. It was therefore not feasible to determine if repeat visits were by the same patient, or if patients were subsequently admitted for prolonged symptoms. This could limit the ability to make inferences on hospitalization rates or recurrent visits in the context of pollution exposure.

### 2.2. Study Participants

Study participants were children aged 2 and 14 who were presented to the pediatric ER and were diagnosed with bronchial asthma by a pediatric emergency physician, as defined by the Global Initiative for Asthma (GINA).

Included criteria: Children aged 2–14 years with confirmed cases of bronchial asthma.

Excluded criteria: Patients with coexisting respiratory conditions (e.g., bronchitis), or whose clinical data are incomplete are excluded.

### 2.3. Environmental Data Collection

Air pollutant concentrations, by day, were collected from National Center for Environmental Compliance:

- Station name: Al-Khobar;
- Station number: S045.

The pollutants studied were as follows:

- PM_2.5_ (fine particulate matter),
- PM_10_ (coarse particulate matter),
- NO_2_ (nitrogen dioxide),
- O_3_ (ozone),
- SO_2_ (sulfur dioxide),
- CO (carbon monoxide).

Meteorological data, including daily temperature and humidity, were also obtained for adjustment in statistical models.

### 2.4. Statistical Analysis

#### 2.4.1. Descriptive Statistics

Daily ER visit counts and environmental variables were summarized using descriptive statistics. Continuous variables were described using means, medians, and interquartile ranges (IQRs), and categorical variables were presented with frequencies and percentages.

#### 2.4.2. Inferential Statistics

- Normality check: Normal distribution assumption was checked; non-parametric methods were applied for skewed distributions.
- Mann–Whitney U test: Differences between ER visits in low pollutant days vs. high pollutant days.
- Kruskal–Wallis test: To compare ED visits in different levels of air pollutants’ concentration ranges.

#### 2.4.3. Correlation and Regression Analysis

Spearman rank correlation was used to examine bivariate correlations between ER visits and air pollutants or meteorological variables. Poisson regression modeling: Adjusted odds ratios (AOR) were calculated for levels of each pollutant and visits to the ER, adjusting for temperature and humidity. The modification effects of temperature and humidity on the PM_2.5_ and PM_10_ asthma association was tested by Interaction Analysis.

#### 2.4.4. Threshold and Time Series Analysis

Threshold analysis identified pollutant concentration levels associated with a significant increase in asthma-related ER visits, using stratified Kruskal–Wallis tests. ARIMA (1,1,2) modeling was used to assess the cyclic patterns and the lag effect (up to 7 days) of air pollution on asthma exacerbations via auto-correlation and partial-autocorrelation functions and analyzed the model parameters.

#### 2.4.5. Significance and Software

All statistics tests were two-tailed, and a *p*-value < 0.05 was considered statistically significant. Analyses were conducted using IBM SPSS Statistics version 27.0.1.

### 2.5. Sample Size and Power Calculation

The minimum required sample size to detect a statistically significant association was 162 pediatric asthma cases (81 per exposure category). The calculation was based on a statistical power > 80% and a 5% margin of error to ensure adequate sensitivity in detecting associations between pollutant exposure and asthma ER visits.

### 2.6. Ethical Considerations

Ethical approval was obtained from Imam Abdulrahman Bin Faisal University IRB (IRB; Approval Number: IRB-2024-01-603). All Patient data were handled in accordance with institutional and ethical standards.

## 3. Results

### 3.1. Descriptive Statistics of ER Visits and Environmental Factors

A total of 1750 pediatric asthma-related ER visits were documented over 366 days during a 12-month study period. Daily ER visits ranged from 0 to 16, with a median of 4 visits per day. The most frequent daily count was four (13.7%), followed by three (13.1%) and two (11.7%). On 55.2% of days, four or fewer visits were recorded, while 44.8% of days had five or more visits.

Regarding air pollutants, the mean nitrogen dioxide (NO_2_) level was 3.86 (SD = 0.37), while ozone (O_3_) had a mean concentration of 137.86 (SD = 86.39), with an interquartile range (IQR) of 78.20 to 185.35. Sulfur dioxide (SO_2_) had an average of 16.80 (SD = 9.84) and an IQR of 11.10 to 22.50. Carbon monoxide (CO) levels averaged 1.90 (SD = 2.59), with a median of 1.25 and an IQR of 0.91 to 1.51. Among particulate matter, PM_2.5_ concentrations had a mean of 76.40 (SD = 55.41), with an IQR of 58.01 to 84.36, while PM_10_ had an average of 0.36 (SD = 0.17) and an IQR of 0.24 to 0.44. Meteorological conditions showed that the mean temperature recorded was 28.15 °C (SD = 7.62), with a median of 28.27 °C and an IQR of 20.65 to 35.74 °C. Humidity levels averaged 44.57% (SD = 17.65), with an IQR of 31.38% to 57.50%. These findings provide insight into the distribution patterns of asthma-related ER visits and their potential associations with air pollution and meteorological conditions (as shown in Table 1; Supplementary Table S2).

### 3.2. Univariate Analysis of Environmental Factors

The univariate analysis revealed significant associations between certain environmental factors and the number of asthma-related emergency room (ER) visits per day. PM_2.5_ levels were significantly higher in the five or more visits group (mean = 77.59; SD = 31.37) compared to the four or fewer visits group (mean = 75.43; SD = 69.10; *p* = 0.029). Conversely, PM_10_ levels were significantly lower in the five or more visits group (mean = 0.33; SD = 0.15) compared to the four or fewer visits group (mean = 0.38; SD = 0.18; *p* = 0.019) (Supplementary Table S1 and Figures S1 and S2). Meteorological factors also showed strong associations with asthma-related ER visits. Temperature was significantly lower in the five-or-more-visits group (mean = 25.78 °C; SD = 6.79) compared to the four-or-fewer-visits group (mean = 30.07 °C; SD = 7.73; *p* < 0.001). Additionally, humidity was significantly higher in the five-or-more-visits group (mean = 50.77%; SD = 15.28) compared to the four-or-fewer-visits group (mean = 39.54%; SD = 17.88; *p* < 0.001). These findings indicate that higher PM_2.5_ levels, lower PM_10_ levels, lower temperature, and higher humidity are significantly associated with an increased number of asthma-related ER visits (Table 2).

### 3.3. Poisson Regression Analysis

This analysis identified several significant environmental factors associated with the number of asthma-related emergency room (ER) visits per day. Nitrogen dioxide (NO_2_) was significantly positively associated with ER visits (B = 0.182; *p* = 0.002; AOR = 1.199; 95% CI: 1.069–1.345), indicating that higher NO_2_ levels increased the likelihood of asthma-related ER visits. Similarly, carbon monoxide (CO) showed a significant positive association (B = 0.036; *p* = 0.006; AOR = 1.036; 95% CI: 1.010–1.063), suggesting that increased CO levels contributed to a higher number of asthma-related ER visits. In contrast, PM_10_ was significantly negatively associated with ER visits (B = −0.729; *p* = 0.001; AOR = 0.482; 95% CI: 0.308–0.756), indicating that higher PM_10_ levels were linked to a lower number of asthma-related ER visits; this may reflect a lack of clear positive association rather than a protective effect. Additionally, humidity demonstrated a strong positive association with asthma-related ER visits (B = 0.018; *p* < 0.001; AOR = 1.019; 95% CI: 1.012–1.025), suggesting that higher humidity levels increased the likelihood of ER visits due to asthma. These findings highlight the role of air pollutants and meteorological factors in influencing asthma exacerbations requiring emergency medical attention (Table 3).

### 3.4. Time-Series Analysis (ARIMA Model)

**Figure 1:**
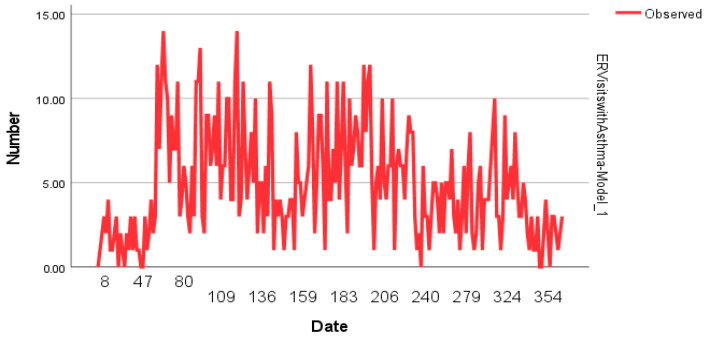
Daily pediatric asthma ER visit counts observed over 366 days. Peaks align with seasonal fluctuations in pollutant exposure and meteorological variables.

The time-series analysis using an ARIMA(1,1,2) model examined the impact of environmental factors on the number of asthma-related ER visits per day. The model demonstrated a stationary R-squared of 0.406, indicating moderate predictive power, while the overall R-squared was 0.297. The root mean square error (RMSE) was 2.763, and the mean absolute percentage error (MAPE) was 70.821, reflecting the variability in asthma-related ER visits. Among the predictors, carbon monoxide (CO) showed a significant influence on asthma-related ER visits. The contemporaneous effect of CO (Lag 0) was positively associated with ER visits (B = 0.450; *p* = 0.014), while its Lag 1 effect was negative (B = −0.461; *p* = 0.012), indicating a possible delayed inverse relationship. Additionally, the Lag 0 effect of CO in the LAGS(CO,2) model was negatively associated with ER visits (B = −0.434; *p* = 0.013), whereas Lag 1 showed a positive association (B = 0.402; *p* = 0.020). The autoregressive (AR) term at Lag 1 was significantly negative (B = −0.662; *p* < 0.001), while the moving average (MA) term at Lag 2 was significantly positive (B = 0.760; *p* < 0.001), highlighting the presence of short-term fluctuations in asthma-related ER visits. The Ljung–Box Q test (χ^2^ = 14.866; *p* = 0.534) suggested that the model residuals were uncorrelated, indicating a good model fit. However, missing values led to the exclusion of PM_10_ lag variables, which may have influenced the overall model performance. These findings suggest that short-term variations in CO levels significantly impact asthma-related ER visits, with both immediate and lagged effects playing a role (Table 4; Figure 1).

### 3.5. Interaction Effects of Pollution with Temperature and Humidity

The Poisson regression analysis identified several significant environmental factors associated with the number of asthma-related emergency room (ER) visits per day. Nitrogen dioxide (NO_2_) was significantly positively associated with ER visits (B = 0.182; *p* = 0.002; AOR = 1.199; 95% CI: 1.069–1.345), indicating that higher NO_2_ levels increased the likelihood of asthma-related ER visits. Similarly, carbon monoxide (CO) showed a significant positive association (B = 0.036; *p* = 0.006; AOR = 1.036; 95% CI: 1.010–1.063), suggesting that increased CO levels contributed to a higher number of asthma-related ER visits. In contrast, PM_10_ was significantly negatively associated with ER visits (B = −0.729; *p* = 0.001; AOR = 0.482; 95% CI: 0.308–0.756), suggesting a lack of positive association rather than a definitive protective effect. Additionally, humidity demonstrated a strong positive association with asthma-related ER visits (B = 0.018; *p* < 0.001; AOR = 1.019; 95% CI: 1.012–1.025), suggesting that higher humidity levels increased the likelihood of ER visits due to asthma. These findings highlight the role of air pollutants and meteorological factors in influencing asthma exacerbations requiring emergency medical attention, as shown in (Table 5).

## 4. Discussion

Our study demonstrates a clear relationship between air pollution and the pediatric asthma exacerbations that require emergency room (ER) visits. Pollutants such as PM_2.5_, NO_2_, and CO were highly correlated with higher ER visits, suggesting their importance in respiratory health compromise. PM_2.5_, due to its fine particulate nature, has a higher penetration rate into the respiratory system because of its small particle size. Vehicular and industrial pollution are the main sources of NO_2_, which has been linked to airway inflammation and enhanced bronchial responsiveness. Similarly, CO may aggravate respiratory distress in susceptible individuals by impairing oxygen delivery. Interestingly, PM_10_ had a negative correlation, indicating that the effect of pollutants might differ according to particle size and other factors.

This study explored the association between ambient air pollution and pediatric asthma-related emergency room visits in the Eastern Province of Saudi Arabia. Data from a total of 366 days were collected, including air pollutant levels and asthma-related ER visits on each day. The results showed that higher PM_2.5_ exposures were strong risk factors for asthma-related ER visits, which may be related to the fine particles’ penetration of the lower respiratory tract, and their influence on inflammation and airway hyperresponsiveness. Conversely, lower PM_10_ levels are related to higher ER visits, likely because there is less exposure to coarse particles trapped by the upper airways, or finer particles such as those fine particulates are preponderant in the high-risk periods.

Meteorological variables played a significant role. Lower ambient temperature and increased humidity were associated with a higher number of ER visits due to asthma. These conditions may enhance the frequency of bronchospasms and allergen exposure. Environmental control is a core component of asthma management, as recommended by the Global Initiative for Asthma (GINA) [9], which emphasizes minimizing exposure to environmental triggers such as air pollutants. Our findings align with this guidance by demonstrating a significant association between air pollution and asthma-related ER visits. Corresponding findings from the US, Europe, and urbanized regions of East Asia regions of East Asia demonstrate the worldwide generalizability of these relationships [10]. Temperature fluctuations were also shown to play a significant role in asthma-related ER visits in Korea [11]. Systematic reviews have highlighted the interaction between temperature and humidity in the seasonal trends of asthma [12].

The findings of this study have significant clinical implications. Understanding that there is an association between air quality and increases in asthma exacerbations can guide clinicians to enhance the quality of care for their patients. Also, educating families and patients about the impact or the crucial role of environmental triggers should be incorporated into the standard of care. Thus, routinely monitoring air quality will aid in reducing the risk of allergen exposure and help prevent further exacerbations. This enables parents to adjust their children’s outdoor activities, particularly on days when air quality metrics reflect elevated levels of NO_2_ or PM_2.5_ [13].

Environmental triggers may not be sufficiently addressed in current asthma care programs in Saudi Arabia, despite increased awareness of the topic. Although the Saudi asthma treatment guidelines emphasize the importance of controlling indoor environmental triggers, there is a lack of clinical incorporation of ambient air quality monitoring [14]. In Jeddah, air pollution is caused by natural sources such as dust storms and local emissions, counting traffic and residual oil combustion from nearby industrial areas [15]. While PM_2.5_ levels consistently exceed WHO standards, these results are representative of the local pollution profile in Jeddah and should not be generalized to all places in Saudi Arabia [15]. Thus, localized source identification is crucial before applying broad policy measures.

A recent meta-analysis showed that both PM_10_ and PM_2.5_ are linked to increased odds of developing asthma and wheezing, with a stronger and more consistent association observed for PM_2.5_ [16]. The review concluded that PM_2.5_ exposure is significantly linked to childhood-onset asthma and wheezing, while PM_10_ showed a weaker or non-significant association [16]. PM_10_ is, on the other hand, made up of larger but less hazardous particles, such as dust and pollen. Furthermore, PM_2.5_ can remain suspended in the air for a longer time, and as the exposure duration increases, the greater the chance of inhalation [17]. Young children are more prone to PM_2.5_ because of their higher air intake per body weight and continuous lung development [18]. Hence, children breathe more air per unit of body weight; children are thus more susceptible to air-borne pollutants, which hinder strong growth and development [18]. These elements, such as PM_2.5_, had low penetration into the deeper lung, higher toxicity, and a more marked inflammatory potential, which possibly explains their closer association with the development of asthma and wheeze in children compared with PM_10_ [19].

Recent research shows that early-life influence of air pollution is integral in the establishment of asthma. A large multicohort study by Zanobetti et al. (2024) [20] showed that PM_2.5_ and NO_2_ during the first 3 years of life were associated with high asthma incidence, in particular, in socially disadvantaged children. Such pollutants may then affect lung and immune function development through oxidative stress and inflammation [20].

Saudi Arabia shows one of the highest mortality risks attributable to fine particulate matter, with each 10 μg/m^3^ increase in PM_2.5_ linked to an estimated 80.3% rise in mortality risk [21]. Integrating the Air Quality Index (AQI) into asthma action plans (AAPs) will lead to better asthma control [22]. Yet, generalizing the results is constrained due to the small size of the sample, and further studies that are large and prospective, conducted in different regions, are required to provide a very complete understanding of the advantages and the possible disadvantages of AQI as an adjunct management tool for asthma [23]. Clinicians can use real-time air quality data to predict and identify the occurrence of sandstorms and acute attacks and to provide guidance to their patients during these episodes [24]. For example, doctors could recommend that preventer medications be used more frequently on days with high NO_2_ or PM_2.5_ levels. The application of these observations allows for more precise identification of children at increased risk for asthma exacerbations, particularly those living in communities with high industrial pollution exposure, enabling them to be targeted with aggressive preventive measures. Children might benefit from increased use of inhaled corticosteroids during periods of high NO_2_ or PM_2.5_.

Past studies have shown robust correlations between PM_2.5_, NO_2_, and O_3_ and asthma-related emergency visits [25,26,27]. Furthermore, although short-term exposure to PM_10_ may be weakly related to asthma ER visits in each location, long-term exposure has also been found to produce persistent airway inflammation and increase asthma risk [6]. These associations are documented across regions in the United States and China, where higher levels of PM_2.5_ and NO_2_ corresponded with increased rates of pediatric asthma visits [25,26,27].

Ultimately, the literature from Saudi Arabia and Europe indicates minimal effects and no significant association between PM_10_ levels and acute asthma presentations following dust storms. This conclusion reinforces our study’s findings, suggesting that pollutants such as PM_2.5_ and NO_2_ may have a more pronounced effect on pediatric asthma compared to larger particles like PM_10_ [28]. However, other studies have shown contradictory results. Reports from Southeast Asia and Europe observed a significant rise in ER visits or hospitalizations from asthma attacks following dust storms with elevated PM_10_ levels [29,30]. However, this discrepancy may be due to regional differences in particle composition, source of PM_10_, or population characteristics. In our study setting, PM_10_ may be less inflammatory due to a higher proportion of coarse natural dust, while PM_2.5_ dominated during periods of high pollution, leading to the observed associations with asthma exacerbations.

Air-quality public alert system alerts and public health advisories are important in reducing the number of asthma-related ER visits by educating the public about environmental triggers. Early warnings of air pollution levels combined with meteorological information can help predict asthma attacks [8]. The warnings can be categorized into four risk levels (low, moderate, high, and very high) depending on pollutant concentrations or weather conditions, with specific information for children with asthma. Especially when pollutants such as PM_2.5_ and NO_2_ are elevated, there should be appropriate risk communication, suggesting remaining indoors during peak periods, ensuring air purifiers.

Furthermore, the real-time index should be instantly available via SMS alerts, social media updates, and mobile apps to help parents decide on their children’s outdoor activities. Moreover, local strategies to protect children are highly advised. Collaborating with the Ministry of Education to address schools is important, as outdoor activities can be modified on days of worsening air quality to minimize students’ exposure to pollutants.

Taken together, these findings highly suggest a close correlation between environmental factors and the rate of asthma-related ER visits. Previous studies have demonstrated the same correlation, showing that exposure to air pollutants such as PM_2.5_, NO_2_, and O_3_ correlates with asthma exacerbations and increased ER visits [25,26]. In addition, the significance of industrial and traffic-related pollutants in aggravating asthma symptoms has been demonstrated, highlighting the need for effective interventions for at-risk populations

Further studies should be conducted to explore the effects of long-term exposure to air pollutants on asthma severity and exacerbation patterns in children. Understanding gene–environment interactions in asthma could help identify children who are potentially at risk from environmental pollutants due to a genetic predisposition [22]. Additionally, this could be an opportunity to investigate possible solutions, such as indoor air filtration devices and city-wide pollutant regulations, as evidence-based methods to reduce the health burdens associated with asthma [31]. To the best of our knowledge, this is the first study conducted in the Eastern Province of Saudi Arabia to demonstrate the association between air pollution and pediatric asthma.

Although in this study only pediatric asthma was studied, other high-risk groups, such as elderly and immunocompromised individuals, should be considered for a better understanding of how air pollution affects the respiratory system, as they might have similar susceptibilities to environmental exposure. The limitations of this study include its retrospective design, which limits the ability to demonstrate a clear causal relationship between exposure to air pollution and asthma exacerbations. Moreover, there are no surveillance data on individual patient exposure to environmental conditions outside the hospital, making it difficult to ascertain actual exposure levels. Also, the dataset lacked information on asthma severity, treatment use, repeat ER visits, and hospital admissions.

## 5. Conclusions

The current data used in this study suggest that air pollution has a high impact on pediatric asthma severity, thus calling for targeted interventions. This study benefits from a large dataset and strong statistical models, and therefore contributes valuable evidence about the relationship between environmental factors and asthma-related hospital visits. However, the retrospective nature and the absence of personal exposure data call for cautious interpretation. Further longitudinal studies, along with research into gene–environment interactions, and the development of targeted interventions are needed to improve asthma management and alleviate its burden among pediatric patients.

## Figures and Tables

**Table 1:** Descriptive statistics of ER visits for asthma and environmental factors.

		N/Mean	%SD	Median	IQR
Number of ER visits with asthma per day	None (0)	20	5.5%		
	1	41	11.2%		
	2	43	11.7%		
	3	48	13.1%		
	4	50	13.7%		
	5	32	8.7%		
	6	36	9.8%		
	7	18	4.9%		
	8	17	4.6%		
	9	18	4.9%		
	10	15	4.1%		
	11	18	4.9%		
	12	5	1.4%		
	13	1	0.3%		
	14	3	0.8%		
	16	1	0.3%		
Number of ER visits per day with asthma	4 or less	202	55.2%		
	5 or more	164	44.8%		
NO_2_		3.86	0.37	3.80	3.80–3.80
O_3_		137.86	86.39	107.05	78.20–185.35
SO2		16.80	9.84	15.10	11.10–22.50
CO		1.90	2.59	1.25	0.91–1.51
PM_2.5_		76.40	55.41	70.63	58.01–84.36
PM_10_		0.36	0.17	0.32	0.24–0.44
Temperature		28.15	7.62	28.27	20.65–35.74
Humidity		44.57	17.65	46.57	31.38–57.50

Data represent daily ER visit counts and summary statistics of environmental and meteorological variables. Units: gases in ppb, PM in µg/m^3^, temperature in °C, and humidity in %.

**Table 2:** Univariate analysis of environmental factors by ER visits for asthma.

	Number of ER Visits per Day with Asthma								
	4 or Less				5 or More				*p*-Value ^U^
	Mean	SD	Median	IQR	Mean	SD	Median	IQR	
NO_2_	3.82	0.18	3.80	3.80–3.80	3.92	0.51	3.80	3.80–3.80	0.097
O_3_	142.33	87.85	107.70	83.20–224.50	132.34	84.51	100.20	72.55–155.83	0.064
SO_2_	16.13	10.44	14.95	10.30–21.00	17.63	9.01	16.20	11.80–23.10	0.088
CO	1.91	2.62	1.25	0.93–1.44	1.88	2.55	1.25	0.90–1.56	0.785
PM_2.5_	75.43	69.10	67.80	53.40–85.09	77.59	31.37	73.26	61.47–82.81	0.029 *
PM_10_	0.38	0.18	0.35	0.26–0.46	0.33	0.15	0.29	0.23–0.41	0.019 *
Temperature	30.07	7.73	32.89	22.14–37.25	25.78	6.79	25.48	19.60–31.80	<0.001 *
Humidity	39.54	17.88	39.51	23.75–51.88	50.77	15.28	52.94	40.57–61.38	<0.001 *

^U^ Independent samples Mann–Whitney U test * *p* < 0.05, significant.

**Table 3:** Poisson regression analysis of environmental factors and ER visits for asthma.

Parameter Estimates										
Parameter	B	Std. Error	95% Wald Confidence Interval		Hypothesis Test			Adjusted Odds Ratio (AOR)	95% Wald Confidence Interval for (AOR)	
			Lower	Upper	Wald Chi-Square	Df	*p*-Value		Lower	Upper
(Intercept)	−0.213	0.4570	−1.108	0.683	0.216	1	0.642	0.809	0.330	1.980
NO_2_	0.182	0.0585	0.067	0.296	9.628	1	0.002	1.199	1.069	1.345
O_3_	0.000	0.0004	0.000	0.001	0.666	1	0.414	1.000	1.000	1.001
SO_2_	0.003	0.0038	−0.005	0.010	0.594	1	0.441	1.003	0.995	1.010
CO	0.036	0.0130	0.010	0.061	7.625	1	0.006	1.036	1.010	1.063
PM_2.5_	0.000	0.0008	−0.002	0.001	0.088	1	0.767	1.000	0.998	1.001
PM_10_	−0.729	0.2292	−1.178	−0.280	10.120	1	0.001	0.482	0.308	0.756
Temperature	0.012	0.0076	−0.003	0.027	2.455	1	0.117	1.012	0.997	1.027
Humidity	0.018	0.0031	0.012	0.024	35.790	1	0.000	1.019	1.012	1.025
(Scale)	1 ^a^									

Dependent variable: number of er visits with asthma per day model: (intercept), NO_2_, O_3_, SO_2_, CO, PM_2.5_, PM_10_, temperature, and humidity. ^a^, Fixed at the displayed value.

**Table 4:** Time-series (ARIMA) lag effects for CO.

Lag	Coefficient (B)	*p*-Value	Interpretation
Lag 0	0.45	0.014	Positive association
Lag 1	−0.461	0.012	Negative association

Lag 1 reflects the next-day effect. Coefficients represent the change in ER visit rates per unit increase in CO.

**Table 5:** Poisson regression analysis of environmental factors.

Variable	AOR	*p*-Value	Interpretation
NO_2_	1.279	<0.001	Significant increase in risk
CO	1.034	0.008	Slight increase in risk
PM_2.5_	0.964	0.001	Inverse effect
PM_2.5_ × temperature	1.001 *	<0.001	PM_2.5_ effect modified by temperature
PM_2.5_ × humidity	1.000 *	0.015	PM_2.5_ effect modified by humidity
PM_2.5_ × temperature	1.001 *	<0.001	PM_2.5_ effect modified by temperature

* Statistically significant at *p* < 0.05.

## Data Availability

The corresponding author will provide the datasets used and/or analyzed during the current study upon reasonable request.

## Supplementary Materials

The following supporting information can be downloaded at https://www.mdpi.com/article/10.3390/jcm14134659/s1, Table S1: Threshold analysis of PM_2.5_ and PM_10_ in relation to asthma ER visits; Figures S1 and S2: Distribution of asthma ER visits across PM_2.5_ and PM_10_ exposure ranges; Table S2: Association between PM_2.5_ and asthma ER visits across humidity and temperature ranges.

## References

[1] (2024). What Is Asthma?. https://www.nhlbi.nih.gov/health/asthma.

[2] World Health Organization: WHO (2024). Asthma. https://www.who.int/news-room/fact-sheets/detail/asthma.

[3] Alahmadi T.S., Banjari M.A., Alharbi A.S. (2019). The prevalence of childhood asthma in Saudi Arabia. Int. J. Pediatr. Adolesc. Med..

[4] Khatri S.B., Newman C., Hammel J.P., Dey T., Van Laere J.J., Ross K.A., Rose J.A., Anderson T., Mukerjee S., Smith L. (2021). Associations of Air Pollution and Pediatric asthma in Cleveland, Ohio. Sci. World J..

[5] Yang X., Zhang Y., Zhan X., Xu X., Li S., Xu X., Ying S., Chen Z. (2021). Particulate matter exposure is highly correlated to pediatric asthma exacerbation. Aging.

[6] Hasunuma H., Takeuchi A., Ono R., Amimoto Y., Hwang Y.H., Uno I., Shimizu A., Nishiwaki Y., Hashizume M., Askew D.J. (2020). Effect of Asian dust on respiratory symptoms among children with and without asthma, and their sensitivity. Sci. Total Environ..

[7] Alangari A., Riaz M., Mahjoub M., Malhis N., Al-Tamimi S., Al-Modaihsh A. (2015). The effect of sand storms on acute asthma in Riyadh, Saudi Arabia. Ann. Thorac. Med..

[8] Bi J., D’sOuza R.R., Moss S., Senthilkumar N., Russell A.G., Scovronick N.C., Chang H.H., Ebelt S. (2023). Acute effects of ambient air pollution on asthma emergency department visits in ten U.S. states. Environ. Health Perspect..

[9] Global Initiative for Asthma Global Strategy for Asthma Management and Prevention. https://ginasthma.org/gina-reports/.

[10] Pfeffer P.E., Mudway I.S., Grigg J. (2020). Air pollution and asthma. CHEST J..

[11] Kim J., Lim Y., Kim H. (2014). Outdoor temperature changes and emergency department visits for asthma in Seoul, Korea: A time-series study. Environ. Res..

[12] Bodaghkhani E., Mahdavian M., MacLellan C., Farrell A., Asghari S. (2019). Effects of Meteorological Factors on Hospitalizations in Adult Patients with Asthma: A Systematic Review. Can. Respir. J..

[13] Ho K., Paez J., Liu B. (2018). Air quality alerts benefit asthmatics. Lancet Planet Health.

[14] Al-Moamary M.S., Alhaider S.A., Allehebi R., Idrees M.M., Zeitouni M.O., Al Ghobain M.O., Alanazi A.F., Al-Harbi A.S., Yousef A.A., Alorainy H.S. (2023). The Saudi initiative for asthma—2024 update: Guidelines for the diagnosis and management of asthma in adults and children. Ann. Thorac. Med..

[15] Lim C.C., Thurston G.D., Shamy M., Alghamdi M., Khoder M., Mohorjy A.M., Alkhalaf A.K., Brocato J., Chen L.C., Costa M. (2018). Temporal variations of fine and coarse particulate matter sources in Jeddah, Saudi Arabia. J. Air Waste Manag. Assoc..

[16] Keleb A., Abeje E.T., Daba C., Tadesse T., Duko B., Mulugeta H., Mohammed Y., Nigatu Y.T. (2025). The odds of developing asthma and wheeze among children and adolescents exposed to particulate matter: A systematic review and meta-analysis. BMC Public Health.

[17] World Health Organization (2021). WHO Global Air Quality Guidelines: Particulate Matter (PM2.5 and PM10), Ozone, Nitrogen Dioxide, Sulfur Dioxide and Carbon Monoxide. Executive Summary.

[18] Pryor J.T., Cowley L.O., Simonds S.E. (2022). The physiological effects of air pollution: Particulate matter, physiology and disease. Front. Public Health.

[19] Yan W., Wang X., Dong T., Sun M., Zhang M., Fang K., Chen Y., Chen R., Sun Z., Xia Y. (2020). The impact of prenatal exposure to PM_2.5_ on childhood asthma and wheezing: A meta-analysis of observational studies. Environ. Sci. Pollut. Res..

[20] Zanobetti A., Ryan P.H., Coull B.A., Luttmann-Gibson H., Datta S., Blossom J., Brokamp C., Lothrop N., Miller R.L., Beamer P.I. (2024). Early-life exposure to air pollution and childhood asthma cumulative incidence in the ECHO CREW Consortium. JAMA Netw. Open.

[21] Isaifan R.J. (2023). Air pollution burden of disease over highly populated states in the Middle East. Front. Public Health.

[22] Tizaoui K., Kaabachi W., Hamzaoui K., Hamzaoui A. (2015). Association of Single Nucleotide Polymorphisms in Toll-like Receptor Genes with Asthma Risk: A Systematic Review and Meta-analysis. Allergy Asthma Immunol. Res..

[23] Rosser F.J., Rothenberger S.D., Han Y.Y., Forno E., Celedón J.C. (2023). Air Quality Index and Childhood Asthma: A Pilot Randomized Clinical Trial Intervention. Am. J. Prev. Med..

[24] Environmental Protection Agency (2013). Near Real Time Modeling of Weather, Air Pollution, and Health Outcome Indicators in New York City.

[25] Rosser F., Han Y.Y., Rothenberger S.D., Forno E., Mair C., Celedón J.C. (2022). Air quality index and emergency department visits and hospitalizations for childhood asthma. Ann. Am. Thorac. Soc..

[26] Vu B.N., Tapia V., Ebelt S., Gonzales G.F., Liu Y., Steenland K. (2021). The association between asthma emergency department visits and satellite-derived PM2.5 in Lima, Peru. Environ. Res..

[27] Ma Y., Yu Z., Jiao H., Zhang Y., Ma B., Wang F., Zhou J. (2019). Short-term effect of PM2.5 on pediatric asthma incidence in Shanghai, China. Environ. Sci. Pollut. Res..

[28] Andersen Z.J., Wahlin P., Raaschou-Nielsen O., Ketzel M., Scheike T., Loft S. (2008). Size distribution and total number concentration of ultrafine and accumulation mode particles and hospital admissions in children and the elderly in Copenhagen, Denmark. Occup. Environ. Med..

[29] Bell M.L., Levy J.K., Lin Z. (2007). The effect of sandstorms and air pollution on cause-specific hospital admissions in Taipei, Taiwan. Occup. Environ. Med..

[30] Andersen Z.J., Wahlin P., Raaschou-Nielsen O., Scheike T., Loft S. (2007). Ambient particle source apportionment and daily hospital admissions among children and elderly in Copenhagen. J. Expo. Sci. Environ. Epidemiol..

[31] Lee G.H., Kim J.H., Kim S., Lee S., Lim D.H. (2020). Effects of Indoor Air Purifiers on Children with Asthma. Yonsei Med. J..

