# Supplementary material for: Evaluating the Impact of Air Quality on Pediatric Asthma-Related Emergency Room Visits in the Eastern Province of Saudi Arabia
Source: J Clin Med. 2025 Jul 1;14(13):4659. doi: 10.3390/jcm14134659 (PMC12251416; doi:10.3390/jcm14134659)

Supplementary

Table S1

Threshold Analysis of Air Pollutants and ER Visits for Asthma

		Number of ER Visits with Asthma Per Day			
		Mean	Median	IQR	P value[K]
PM2.5 Range	0-12	1.90	2.00	1.00-3.00	<0.001*
	12.1-35.4	2.00	2.00	2.00-2.00	
	35.5-55.4	4.92	4.00	3.00-6.00	
	55.5-150.4	5.15	4.00	3.00-7.00	
	150.5-250.4	4.67	6.00	2.00-7.00	
	250.5-Higher	1.67	1.50	.00-3.00	
PM10 Range	0-54	.	.	.-.	0.062
	55-154	4.25	3.50	3.00-5.50	
	155-254	5.72	5.00	4.00-7.50	
	255-354	4.80	4.00	2.00-6.00	
	355-424	4.51	4.00	2.00-7.00	
	425-Higher	4.39	3.00	2.00-6.00	

[K]Independent Samples Kruskal-Wallis test
*p<0.05, Significant

Table S2

Impact of $PM_{2.5}$ and PM_{10} on Asthma-Related ER Visits Across Humidity and Temperature Ranges

				Number of ER Visits with Asthma Per Day			
				Mean	Median	IQR	P value[K]
Humidity Ranges	<20%	*PM2.5* Range	0-12	1.67	1.50	1.00-2.00	0.210
			12.1-35.4	.	.	.-.	
			35.5-55.4	5.00	5.00	5.00-5.00	
			55.5-150.4	1.62	1.00	.00-2.00	
			150.5-250.4	.00	.00	.00-.00	
			250.5-Higher	2.50	2.50	2.00-3.00	
	20-50%	*PM2.5* Range	0-12	2.06	2.00	1.00-3.00	<0.001*
			12.1-35.4	2.00	2.00	2.00-2.00	
			35.5-55.4	4.42	4.00	3.00-6.00	
			55.5-150.4	4.95	4.00	3.00-7.00	
			150.5-250.4	6.00	6.00	6.00-6.00	
			250.5-Higher	.00	.00	.00-.00	
	>50%	*PM2.5* Range	0-12	.	.	.-.	0.221
			12.1-35.4	.	.	.-.	
			35.5-55.4	5.23	5.00	3.00-7.00	
			55.5-150.4	6.12	6.00	4.00-9.00	
			150.5-250.4	5.14	6.00	2.00-8.00	
			250.5-Higher	2.50	2.50	1.00-4.00	
Humidity Ranges	<20%	PM10 Range	0-54	.	.	.-.	0.452
			55-154	.	.	.-.	
			155-254	2.67	2.50	2.00-3.00	
			255-354	1.50	1.00	1.00-2.00	
			355-424	1.80	2.00	1.00-3.00	
			425-Higher	1.83	2.00	1.00-2.00	
	20-50%	*PM10* Range	0-54	.	.	.-.	0.652
			55-154	4.43	4.00	3.00-6.00	
			155-254	5.43	5.00	3.00-7.00	
			255-354	4.42	4.00	2.00-6.00	
			355-424	4.87	4.00	2.00-7.00	
			425-Higher	4.26	3.00	2.00-6.00	
	>50%	*PM10* Range	0-54	.	.	.-.	0.130
			55-154	4.00	3.00	3.00-4.00	
			155-254	6.45	6.00	5.00-8.00	
			255-354	6.23	5.50	4.00-10.00	
			355-424	4.95	4.00	3.00-7.00	
			425-Higher	5.07	4.00	2.00-7.50	
Temperature Ranges	<25 C	*PM2.5* Range	0-12	.	.	.-.	0.431
			12.1-35.4	2.00	2.00	2.00-2.00	
			35.5-55.4	5.43	5.00	3.50-7.50	
			55.5-150.4	5.42	5.00	3.00-7.00	
			150.5-250.4	4.86	6.00	2.00-7.00	
			250.5-Higher	2.50	2.50	1.00-4.00	

	25-35 C	*PM2.5* Range	0-12	3.00	3.00	3.00-3.00	0.017*
			12.1-35.4	.	.	.-.	
			35.5-55.4	4.27	4.00	3.00-6.00	
			55.5-150.4	6.40	6.00	4.00-9.00	
			150.5-250.4	8.00	8.00	8.00-8.00	
			250.5-Higher	.	.	.-.	
	>35 C	*PM2.5* Range	0-12	1.86	2.00	1.00-3.00	0.225
			12.1-35.4	.	.	.-.	
			35.5-55.4	.	.	.-.	
			55.5-150.4	3.14	2.00	1.00-4.00	
			150.5-250.4	.00	.00	.00-.00	
			250.5-Higher	1.25	1.00	.00-2.50	
Temperature Ranges	<25 C	*PM10* Range	0-54	.	.	.-.	0.359
			55-154	4.80	4.00	3.00-6.00	
			155-254	5.75	5.00	4.00-7.00	
			255-354	6.18	5.50	3.00-10.00	
			355-424	4.23	4.00	1.00-6.00	
			425-Higher	4.81	3.50	2.50-7.50	
	25-35 C	*PM10* Range	0-54	.	.	.-.	0.491
			55-154	3.00	3.00	3.00-3.00	
			155-254	6.50	6.00	4.50-9.00	
			255-354	5.45	4.50	4.00-6.00	
			355-424	6.33	5.00	3.00-10.00	
			425-Higher	4.95	4.00	3.00-6.00	
	>35 C	*PM10* Range	0-54	.	.	.-.	0.480
			55-154	.00	.00	.00-.00	
			155-254	4.00	2.50	2.00-5.00	
			255-354	2.24	2.00	1.00-3.00	
			355-424	2.36	2.00	1.00-3.00	
			425-Higher	3.73	2.00	1.00-5.00	

[K]Independent Samples Kruskal-Wallis test

*p<0.05, Significant

Figure S1

Scatter Plot of PM2.5 and Asthma-Related ER Visits

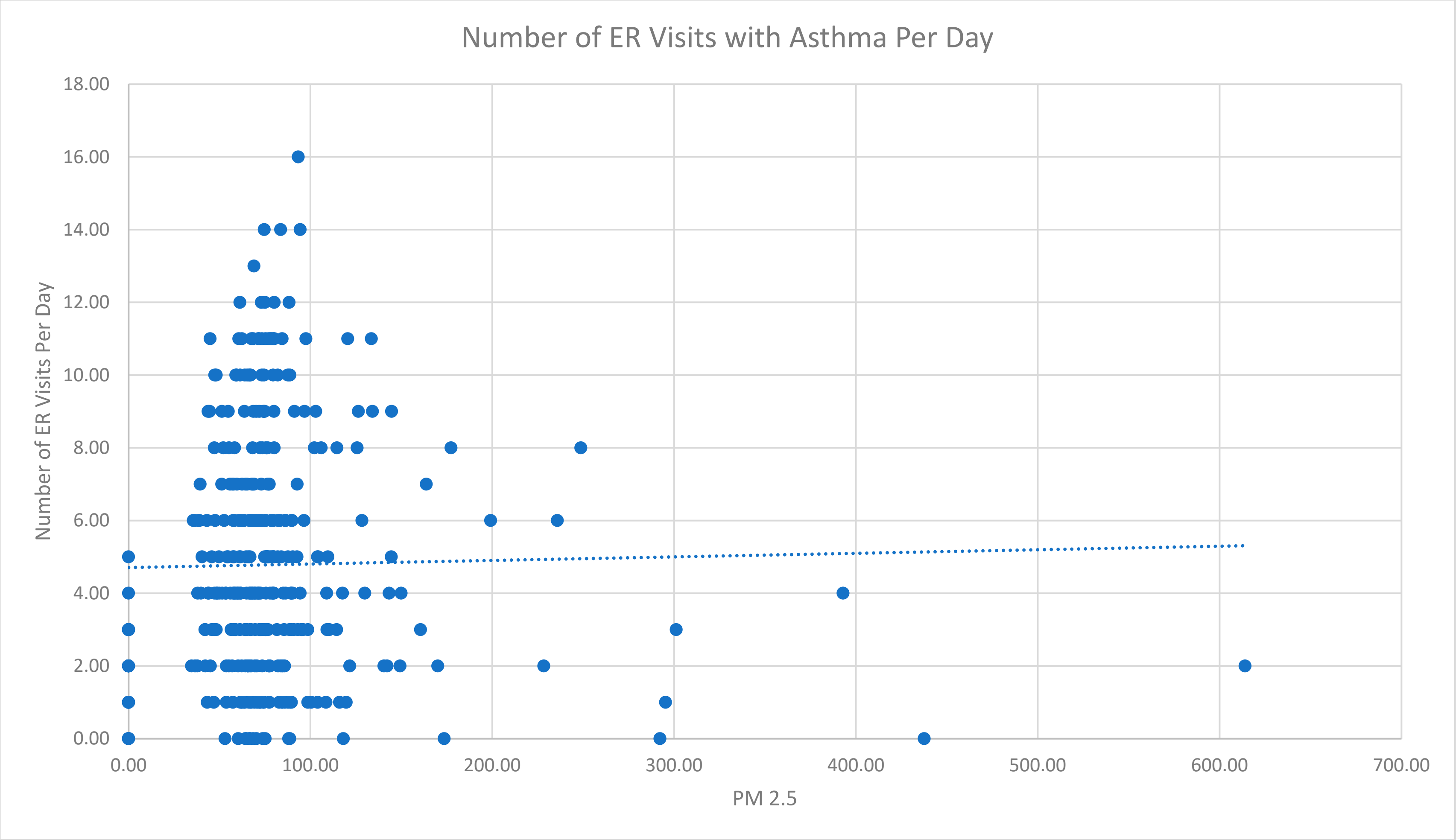

Figure S2

Scatter Plot of PM10 and Asthma-Related ER Visits

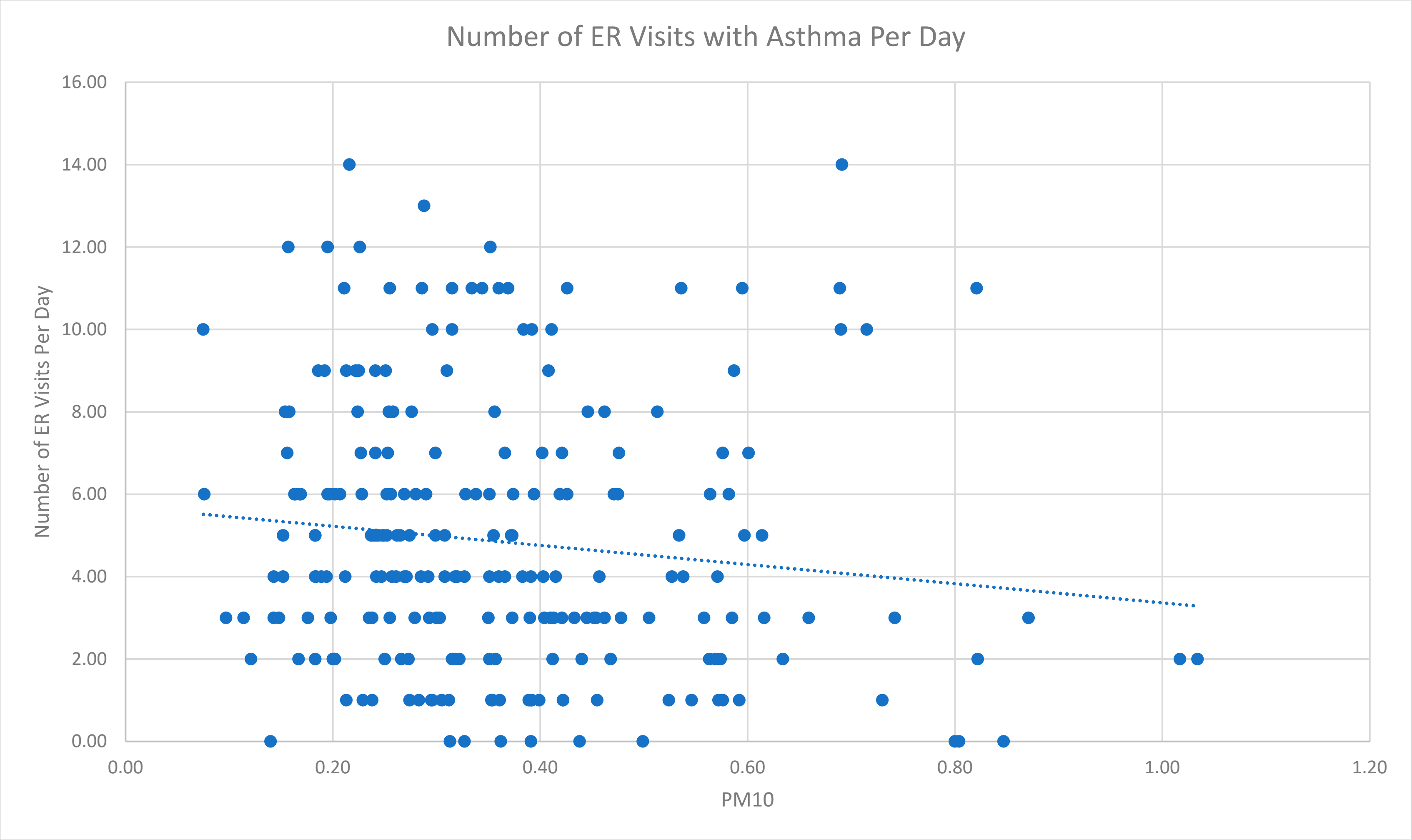

Supplement: Supplementary file 1 [file jcm-14-04659-s001.zip › jcm-3679406-supplementary.pdf]
